# Machine learning-assisted single-cell Raman imaging for rapid, sensitive detection and intracellular mapping of carotenoids in plant cell cultures

**DOI:** 10.1007/s00299-026-03858-x

**Published:** 2026-05-23

**Authors:** Bárbara A. Rebelo, Ensieh Iranmehr, Begoña Espiña, Laura Rodriguez-Lorenzo, Rita Abranches

**Affiliations:** 1 Instituto de Tecnologia Química E Biológica António Xavier (ITQB NOVA), Universidade Nova de Lisboa, 2780-157 Oeiras, Portugal; 2 International Iberian Nanotechnology Laboratory, Av. Mestre José Veiga, 4715-330 Braga, Portugal

**Keywords:** Tobacco BY-2 cells, Intracellular localization, Astaxanthin, Canthaxanthin, Automated Raman spectra analysis, Label-free identifi cation

## Abstract

**Key message:**

CRaman imaging combined with a multi-layer perceptron neural network enables non-destructive, label-freeclassifi cation of tobacco BY-2 cells based on carotenoid composition.

**Abstract:**

Carotenoids are natural tetraterpenoid pigments with important nutritional properties and broad industrial applications. Enhancing their production in plant-based biofactories offers a sustainable alternative to current manufacturing processes. In this work, we developed a label-free, single-cell analytical platform combining Raman imaging with a multi-layer perceptron neural network to classify tobacco BY-2 cells based on their carotenoid content. Carotenoid standards analysis, including astaxanthin, canthaxanthin, and β-carotene, was performed by surface-enhanced Raman scattering using hydrophobic gold nanostars due to the low concentration available. This analysis allowed the assignment of characteristic Raman peaks, specifically at 1160 cm^−1^ and 1520 cm^−1^, of key carotenoids and their identification inside of the cells by Raman imaging. The Raman fingerprints were correlated with carotenoid profiles obtained by HPLC, enabling accurate differentiation between wild-type and transgenic cell lines. In the analyzed transgenic lines, carotenoids accumulated in vesicle-like structures near the nucleus and along the cytoplasmic membrane. This method provides a non-destructive, label-free approach with high classification accuracy and sorting potential based on carotenoid composition, and may be a useful tool for plant synthetic biology and metabolic engineering.

**Supplementary Information:**

The online version contains supplementary material available at 10.1007/s00299-026-03858-x.

## Introduction

Carotenoids are a diverse class of pigments found ubiquitously in nature, known for their vibrant colors ranging from yellow to red (Rodriguez-Concepcion et al. 2018). Structurally, carotenoids comprise a 40-carbon backbone composed of eight isoprene units, often forming linear, monocyclic, or bicyclic structures. Their long conjugated double-bond systems enable visible light absorption, essential for photosynthesis and photoprotection (Namitha and Negi 2010; Ruiz-Sola and Rodríguez-Concepción, 2012; Rebelo et al. 2020a). In addition to their biological functions, carotenoids are valuable in the nutraceutical, pharmaceutical, cosmetic, animal feed, and textile dye industries (Zhu et al. 2009; Rebelo et al. 2020a; Stra et al. 2023).

The biosynthesis of carotenoids has been extensively studied, and genetic engineering has made carotenoid production more sustainable and economically viable. In our previous research, ketocarotenoid production was successfully established in undifferentiated, non-photosynthetic platforms, specifically *Nicotiana tabacum* BY-2 and *Medicago truncatula* A17 cultured cells (Gomes et al. 2025; Rebelo et al. 2025). Typically grown in the dark, these cells became etiolated, a state characterized by the loss of plastid functionality. Even though they lack differentiated chloroplasts or chromoplasts (organelles typically essential for carotenoid storage), astaxanthin and canthaxanthin, along with other carotenoids, were successfully produced through metabolic engineering. However, the exact subcellular structures in which these pigments accumulate remain unclear (Rebelo et al. 2025).

In plants, carotenoids are stored in various types of plastids, each with distinct functions and storage capacities (Cazzonelli and Pogson 2010; Sun et al. 2018). In non-photosynthetic tissues, carotenoids can accumulate in leucoplasts or in amyloplasts, a type of leucoplast specialized in starch storage (Sun et al. 2018). In chloroplasts, primary carotenoids are involved in light harvesting and photoprotection, while secondary carotenoids are metabolized under stress and not essential for photosynthesis (Lichtenthaler 1987; Collins et al. 2011). It has been demonstrated that the stress-induced synthesis of reactive oxygen species (ROS) stimulates the production of secondary carotenoids, which act as antioxidants to reduce ROS levels (Goiris et al. 2012; Rebelo et al. 2020a). The highest carotenoid accumulation typically occurs in chromoplasts, specialized plastids found in flowers, fruits, and senescent leaves, where carotenoids are stored in sub-organellar structures such as globular, crystalline, membranous, fibrillar or tubular forms (Schaub et al. 2018; Zheng et al. 2020).

Various advanced chromatographic techniques have been developed to separate and identify carotenoids, including thin-layer chromatography (TLC), high-performance liquid chromatography (HPLC), or liquid chromatography-tandem mass spectrometry (LC–MS/MS). Although these methods provide reliable results (Rivera and Canela-Garayoa 2012; Jehlička et al. 2014b; Radu et al. 2016), the sample preparation process is time-consuming, making them less suitable for rapid and effective detection. Optical methods like ultraviolet–visible spectroscopy (UV–Vis), on the other hand, can be easily interfered with by other chromophores in the sample, leading to inaccurate detection of the real signal (Rivera and Canela-Garayoa 2012). Chemical fingerprint-based spectroscopic techniques, such as Raman spectroscopy and surface-enhanced Raman scattering (SERS), have been considered as alternative tools for rapid, accurate analysis of carotenoids.

Raman spectroscopy has become one of the most utilized spectroscopic techniques. It is a technique that utilizes inelastic scattering, a phenomenon in which photons from an incident monochromatic light with a certain frequency interact with a molecule, and a very small fraction of this light is scattered, resulting in a frequency shift (to higher or lower frequencies) that provides molecular vibrational information (Jehlička et al. 2014a; Caldwell et al. 2023). It is fast and nondestructive and generally requires simpler sample preparation. This method provides detailed information about molecular structure, crystallinity, and interactions (Ryabchykov et al. 2018; Caldwell et al. 2023). It is particularly sensitive for detecting polyenes (characteristic poly-unsaturated organic compounds), and this sensitivity is further enhanced when using excitation wavelengths close to 500 nm, where resonance effects amplify the signal (Llansola-Portoles et al. 2017). It also enables in vivo analysis of single cells, where the Raman fingerprint reflects biochemical composition and physiological state. Combined with optical imaging, Raman allows spatial mapping of molecules such as carotenoids inside the cell, making it advantageous for direct in vivo observations (Jehlička et al. 2014a).

Despite its advantages, conventional Raman spectroscopy is limited by an inherently weak signal intensity and noise interference. SERS overcomes these limitations by amplifying Raman signal up to 10^14^ orders of magnitude when target molecules are close to plasmonic nanostructures, typically gold and silver (Li et al. 2012; Caldwell et al. 2023). Its sensitivity depends on the development of very active SERS substrates to be able to achieve single-molecule detection. Due to their unique morphology, gold nanostars (GNSs) effectively concentrate electromagnetic fields at their tips, leading to significant signal amplification and improved detection sensitivity (Rodríguez-Lorenzo et al. 2010, 2011).

Over the past decade, Raman spectroscopy has been employed to detect carotenoids in various biological samples. In microalgae, Raman microscopy has been applied to identify and analyze carotenoids, providing information about their biosynthesis and accumulation (Huang et al. 2010; Abbas et al. 2011; Zhong et al. 2021). Similarly, ongoing research is exploring the use of Raman-based methods to investigate carotenoid presence and function in higher plants, further expanding our understanding of these pigments (de Oliveira et al. 2010; Brackmann et al. 2011; Gierlinger et al. 2012; Mateu et al. 2016). It enables researchers to monitor pigment localization, assess biosynthetic dynamics, and explore storage patterns in various plastid types. By integrating SERS and confocal Raman microscopy, researchers have achieved sensitivity and specificity in detecting and studying important biomolecules (Cintə Pinzaru et al. 2015; Radu et al. 2016).

In this study, we applied Raman imaging combined with machine learning (ML) models to investigate the intracellular localization and identification of carotenoids in *Nicotiana tabacum* BY-2 cells. We also used this approach to discriminate between wild-type (WT) and metabolically engineered BY-2 cell lines (W04, YW02, IW09, YIW6, and YIW135). To overcome the limitation of conventional Raman mapping analysis, which is slow and labor-intensive (Qi et al. 2023), we developed a pipeline that combines preprocessing techniques and machine learning algorithms. This approach enables sensitive and label-free identification of carotenoids in undifferentiated living plant cells, provides insights into their intracellular distribution, and supports a high-throughput tool for studying carotenoid accumulation in plant-based expression platforms.

## Materials and methods

### Plant material

*Nicotiana tabacum* BY-2 cultured cells were maintained as previously described, with minor modifications (Rebelo et al. 2020b, 2025). Briefly, these cells were grown in supplemented MS medium at 28 °C under a 16-h light and 8-h dark photoperiod, with a light intensity of 80–100 µmol m^−2^ s^−1^. Wild-type (WT) cell suspension cultures were maintained either in the dark (hereafter referred to as WT-D) or under the specified photoperiod (referred to as WT-L). Additionally, metabolically engineered BY-2 cell lines (W04, YW02, IW09, YIW6, and YIW135), which produce carotenes, xanthophylls, and/or ketocarotenoids were selected for this study (Rebelo et al. 2025).

### Microscopic observations of starch content

To evaluate starch accumulation, BY-2 WT-L cells were initially grown in MS medium supplemented with 0.1 and 1 µg mL^−1^ of kinetin to promote carotenoid production. Following a three-day period, WT-L cells were transferred to MS medium devoid of the synthetic auxin 2,4-D to induce differentiation of starch-storing cells (Miyazawa et al. 2002b). Aliquots were collected 24 and 48 h post-transfer and the cells were stained with Lugol solution (5% of iodine and 10% of potassium iodide in water), which had been filtered and stored in the dark. Bright-field images with different magnifications were acquired in a Leica DMRB microscope equipped with a DFC340 FX digital camera.

### Analysis of carotenoid profile in BY-2 cells

Total carotenoid extraction and quantification were performed as described by Rebelo et al. (2025). Briefly, lyophilized cells were incubated with a Hex:EtOAc mixture for 2 h at 45 °C. The process was repeated until color exhaustion. The combined organic phases were evaporated, and the residue was resuspended in methanol. Carotenoids were separated by HPLC using a linear acetonitrile–water gradient and detected at 445 nm. Identification was based on retention time and UV–Vis spectra compared to authentic standards, and quantification was achieved by peak integration.

### Surface-enhanced Raman spectroscopy analysis of pure carotenoids

#### Preparation of 1-dodecanethiol-functionalized gold nanostars (DDT-GNSs)

All glass labware was cleaned with *aqua regia* (1:3 (v/v) ratio of 64% HNO_3_: 37% HCl) and Milli-Q® ultrapure water (18.2 MΩ.cm) prior to GNSs synthesis. The gold nanostars were synthesized as previously described (Rodríguez-Lorenzo et al. 2019). Briefly, the spherical citrate-coated gold nanoparticles (AuNPs, 15 nm diameter) were prepared by the Turkevich method (Turkevich et al. 1951), and further used as seeds for creating GNSs. The citrate coating of these AuNPs seeds was replaced by polyvinylpyrrolidone (PVP, TCI) by adding 1 mL of PVP (5.6 mM, Mw = 10,000 g mol^−1^; 60 molecules PVP per nm^2^) to 25 mL of AuNPs ([Au] = 0.6 mM) under stirring at RT. The mixture was incubated overnight, and the resulting dispersion was centrifuged for 90 min at 8 000 g and redispersed in ethanol. Then, the GNSs were prepared using the seed-mediated growth method. Briefly, PVP was dissolved in dimethylformamide to a concentration of 10 mM, mixed with an aqueous solution of HAuCl_4_ and the mixture ([Au] = 0.5 mM) was vigorously stirred for 1 min 45 s, allowing full reduction of Au^3+^ to Au^1+^. Immediately after, the PVP-coated AuNPs were added to the reaction mixture, which was stirred for at least 30 min at RT. The resulting GNSs were purified by centrifugation (3 times at 3 500 g for 35 min) and redispersed in water, followed by storage at 4 °C in dark conditions until further use.

To enhance carotenoid interaction, GNSs were functionalized with 1-dodecanethiol (DDT, Sigma-Aldrich). For this, GNSs were transferred to isopropanol (centrifugation at 3 500 g for 35 min), reaching a concentration of 0.43 mM. Then, 12 mL of DDT in isopropanol (2.5 µM) was mixed with 12 mL of GNSs in isopropanol (0.43 mM) under stirring. The mixture was incubated for 4 h, centrifuged (3 500 g for 30 min) and the resulting pellet was dispersed in methanol to a final concentration of 1.7 mM. The GNSs were characterized by transmission electron microscopy (TEM) and ultraviolet–visible-near infrared (UV–Vis-NIR) spectroscopy.

#### SERS samples preparation and analysis

Prior to SERS measurements, the GNSs ([Au] = 1.7 mM) were concentrated approximately 12-fold to achieve a final concentration of 20 mM. Then, 1.5 µL of methanolic solutions of the analytical standards, astaxanthin (250 µg mL^−1^), canthaxanthin (62.5 µg mL^−1^), and β-carotene (62.5 µg mL^−1^) were added separately to 1.5 µL of concentrated GNSs (20 mM). The carotenoid-loaded solutions were released from the micropipette tip by immersing the tip into the colloidal GNSs previously placed on a microscope glass slide. Samples were left to dry overnight at RT before SERS measurements. SERS spectra of the carotenoid-conjugated GNSs were acquired using a WiTec Alpha 3000 R confocal Raman microscope, with a high-resolution diffraction grating (600 gr/mm) and a built-in CCD camera for bright-field imaging. The laser spot (785 nm laser line and a power of 1.8 mW) was focused using 20 × and 50 × objectives. The SERS signals were recorded using 0.1 s integration time and 30 scans per spectrum. Ten spectra were recorded for each sample. Data were processed using Witec software Project 5.2 (cosmic ray removal and baseline correction).

### Raman mapping of wild-type and metabolically engineered BY-2 cell lines

Tobacco BY-2 WT-D, WT-L, W04, YW02, IW09, YIW6, and YIW135 cells were collected during the exponential growth phase, placed on a microscope slide, and covered with a glass coverslip. Bright-field images of were collected using the same system as for Raman acquisition, a WiTec alpha300R confocal Raman microscope with a 50 × objective (Zeiss EC Epiplan 50 × / 0.7; N.A. 0.55, laser spot size of ca. 2 µm). Raman maps were acquired upon excitation with a 532 nm laser line and a selected grating of 600 lines mm^−1^. The cells were manually and rapidly scanned to identify prominent Raman signals associated with carotenoids. The mapped area was selected according to the cell's position on the slide; therefore, the map size and number of spectra per Raman varied. To achieve enhanced signal-to-noise ratios, the Raman signal was monitored in real-time following laser incidence on selected areas of the cell before mapping the region of interest. Details about the XY dimension and the number of spectra used for each map are included in Table S1. Raman maps were collected with integration times of 0.5 s per spectrum, with a step size of 1 µm, and the laser power at the surface of the sample adjusted to 1.8 mW. Bright-field images of each cell were captured before Raman data acquisition to document the initial state of the cells. Post-acquisition, the cells were re-examined to confirm their integrity was maintained throughout the procedure. All Raman spectra were exported to.txt format for ML-assisted data analysis using WITec Control 5.2 software. The Raman map images were obtained by selecting a representative peak of the carotenoids, corresponding to the ν(C = C) vibrational mode within the 1500–1560 cm^−1^ spectral window. Bright-field and Raman map images were processed using WITec Control 5.2 software. The final images were prepared using Affinity Photo (v1.10.5.1342).

### Automated Raman spectra analysis for the identification of carotenoids in plant cells

#### Spectroscopic datasets

**Fig. 1:**
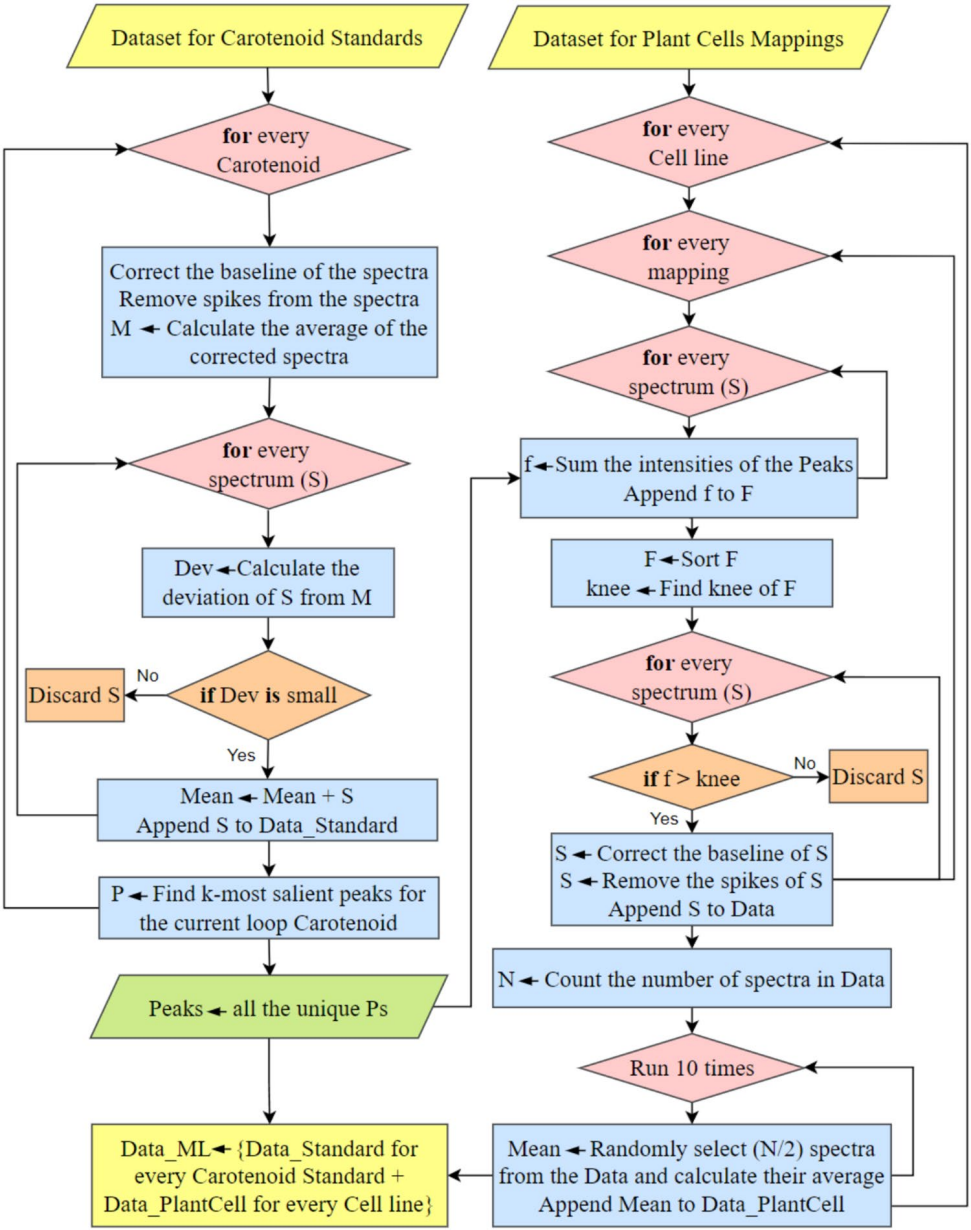
Flowchart of the proposed data preparation method for the machine learning model

**Fig. 2:**
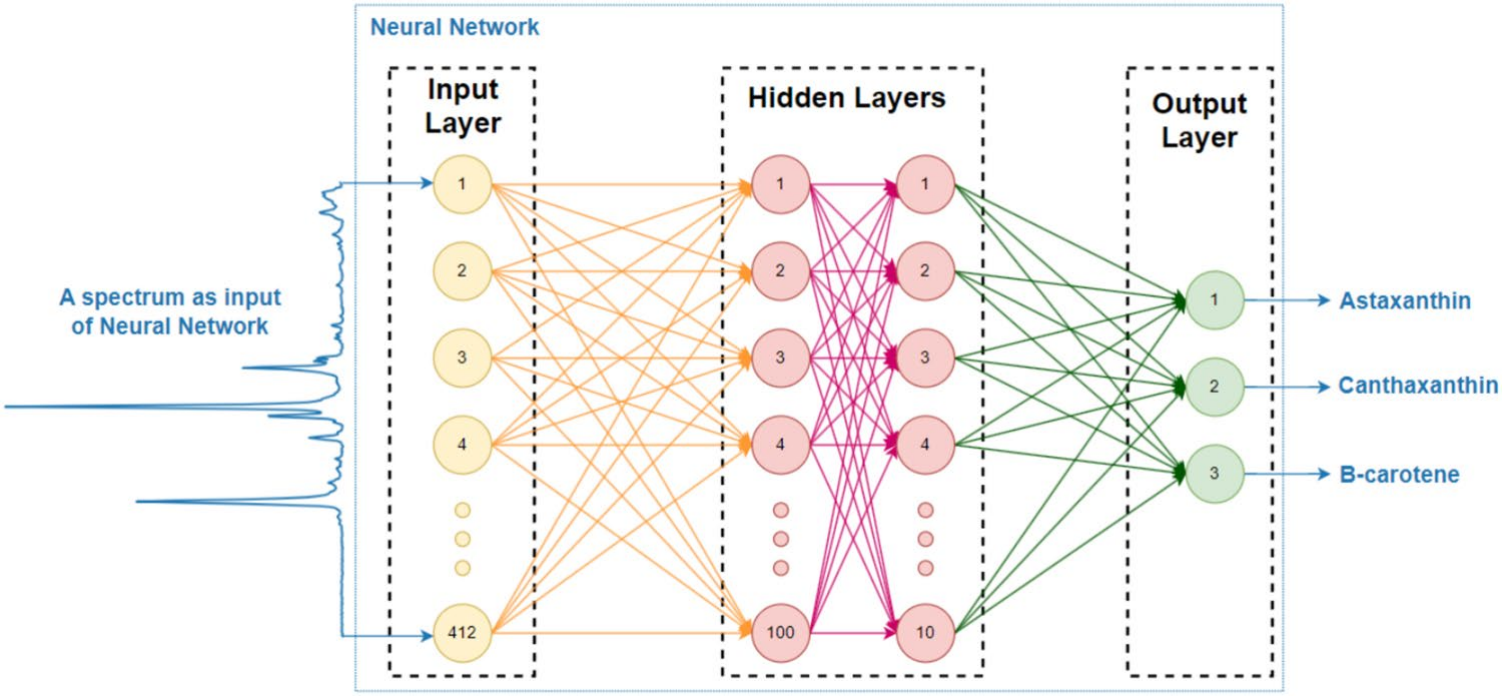
A Multi-Layer perceptron neural network architecture designed for predicting the percentage of each carotenoid standard

Two primary sources of data were used to establish the analysis pipeline. Carotenoid standards dataset includes Raman spectra for three carotenoids: astaxanthin, canthaxanthin, and β-carotene. The SERS spectra of each carotenoid were acquired as previously described. A limited number of spectra represent each carotenoid sub-dataset and Table S2 summarizes both the total number of Raman spectra experimentally acquired and the number of informative Raman spectra (i.e., spectra that contain the characteristic peaks of each compound). The second dataset consisted of Raman mapping of tobacco BY-2 cell lines, comprising single-cell maps from seven cell lines (WT-D, WT-L, W04, YW02, IW09, YIW6, and YIW135). Multiple maps were acquired for each cell line, which contain different spectra numbers (see Table S1 and experimental details in the section “Raman mapping of wild-type and metabolically engineered BY-2 cell lines”). A filtering method was applied to remove non-informative spectra, retaining only those that provide relevant information about the carotenoids, as illustrated in Fig. 1. In this context, non-informative spectra refer to those lacking the Raman fingerprint of carotenoids. Table S1 details the number of maps per cell line, the dimensions of each map, and the number of informative spectra extracted for each map. To train and evaluate the machine learning model used in this work (Figs. 1 and 2), the percentages of synthesized carotenoids in the cell estimated by HPLC (Table S3) were used as labels for the detection of these three carotenoids. It should be noted that HPLC measurements represent population-level averages across a large number of cells and therefore do not capture cell-to-cell metabolic heterogeneity. Consequently, the model was trained to learn population-informed spectral patterns rather than absolute carotenoid concentrations at the single-cell level.

#### Data preparation for the machine learning model

The step-by-step process of preparing the data for training and evaluating our machine learning model, utilizing both the carotenoid standards dataset and the single-cell Raman mapping dataset, is illustrated in Fig. 1. It shows a comprehensive diagram of the data preparation process for both datasets to enhance clarity. We first used the SERS fingerprints of carotenoid standards to identify the k-most important characteristic peaks (i.e., the characteristic peaks observed in the SERS spectra). These peaks serve as filters to remove non-informative spectra from the single-cell BY-2 Raman maps. The spectra of carotenoid standards were processed by applying baseline correction using arPLS (Baek et al. 2015) and spike removal using the Whitaker-Hayes method (Whitaker and Hayes 2018). Subsequently, the average of the corrected spectra was calculated. Spectra that deviated significantly from the average were discarded to retain less noisy spectra for each carotenoid standard. This process resulted in a total of 34 spectra for the carotenoid standards dataset. Finally, the k-most important peaks were identified, representing characteristic carotenoid peaks. Using the carotenoid standards dataset, the process highlighted the 2-most important peaks centered at 1160 cm^−1^ and 1520 cm^−1^.

Using the 2-most important peaks, each cell line’s spectra were clustered into two distinct groups: one containing information relevant to the carotenoid standard, and the other containing non-informative spectra lacking such signals. To distinguish between the two groups, the intensities at 1160 cm^−1^ and 1520 cm^−1^ were summed for each spectrum per cell line map, resulting in a value (f) for each spectrum, that were then placed into a vector (F). By sorting F and identifying the knee point on the F curve, the spectra were clustered into informative and non-informative spectra groups. For each cell line, all the informative spectra (i.e., spectra containing the 2-most important peaks) from different maps were considered as the relevant data. Due to spectral variability and noise, the number of spectra in each cell line (N) was counted, and half randomly selected to calculate an average spectrum. This process was repeated ten times, resulting in ten representative spectra per cell line. From the seven independent cell lines, this process resulted in a total of 70 spectra for the Raman mapping dataset.

#### Machine learning model for carotenoid identification

Before feeding the data to the neural network, all spectra were interpolated to ensure they had the same number of features (i.e., the same number of data points per spectrum). For standardization, a MinMaxScaler was used to scale each feature to a specified range, thereby enhancing the algorithm’s performance. The dataset was then split into training and testing subsets using Multi-label stratified KFold cross-validation, to maintain the distribution of label combinations across folds.

The supervised model used for predicting carotenoid percentages was a Multi-Layer Perceptron regressor (MLPRegressor), a feed-forward neural network with the structure of (${N}_{f}\times ({N}_{h1}\times {N}_{h2})\times {N}_{o}$) (Fig. 2). The number of neurons in the input layer was (${N}_{f}=412$), corresponding to the number of features in each spectrum. The network included two hidden layers with (${N}_{h1}$=100) and (${N}_{h2}$=10) neurons, respectively, followed by an output layer with (${N}_{o}$=3) neurons, representing the three carotenoid standards.

Backpropagation was employed to train the neural network using the “L-BFGS” (Limited-memory Broyden–Fletcher–Goldfarb–Shanno) optimizer, a type of second-order optimization algorithm. For comparison, results using the “ADAM” (Adaptive Moment Estimation) optimizer were also included. Additionally, the “tanh” activation function was used for the hidden layers.

Model performance was evaluated using Mean Square Error (MSE), Mean Absolute Error (MAE), and R^2^ Score. If the dataset had (N) observations, the MSE, MAE, and R^2^ Scores were calculated using Eqs. 1, 2, and 3, respectively. The best possible R^2^ Score is 1, indicating perfect prediction. However, the R^2^ Score can be negative, as the model can perform arbitrarily worse than a simple mean prediction. The optimal result is achieved when the R^2^ Score is closer to 1, and both the MSE and MAE are closer to 0.

1 $$MSE= \frac{1}{N}\sum_{i=1}^{N}{{(yTrue}_{i}-{yPred}_{i})}^{2}$$

2 $$MAE= \frac{1}{N}\sum_{i=1}^{N}|{yTrue}_{i}-{yPred}_{i}|$$

3 $$R2=1- \frac{{\sum}_{i=1}^{N}{{ (yTrue}_{i}-{yPred}_{i})}^{2}}{{\sum}_{i=1}^{N}{{ (yTrue}_{i}-\overline{yTrue })}^{2}}, \overline{yTrue }=\frac{1}{N}\sum_{i=1}^{N}{ yTrue}_{i}$$

## Results and discussion

### Differentiation of BY-2 amyloplasts led to starch accumulation

Since BY-2 cells do not contain fully developed carotenoid-storing organelles, such as chloroplasts or chromoplasts, our main objective was to elucidate the intracellular localization of carotenoid accumulation in wild-type and engineered *Nicotiana tabacum* BY-2 cells. Initial observations using transmitted light microscopy confirmed that wild-type cells grown under controlled light conditions (WT-L) maintained their overall morphology, comparable to the wild-type cells grown in the dark (WT-D) (Figure S1). Important cellular structures, including the nucleus, cell wall, and vacuole, were intact, indicating preserved integrity. Over time, however, apparent vesicle-like structures were detected in WT-L cells exposed to a 16-h photoperiod. To further investigate the localization of carotenoid accumulation, we modified the growth media formulation to induce plastid differentiation into amyloplast (Sakai et al. 1992; Miyazawa et al. 2002a). WT-L cells were first cultivated in MS medium supplemented with kinetin and then transferred to supplemented MS medium deprived of the auxin 2,4-D. Within 24–48 h, we observed a decline in cell proliferation, which was consistent with the expected response to an auxin-cytokinin imbalance, shifting the cells from division to differentiation.

**Fig. 3:**
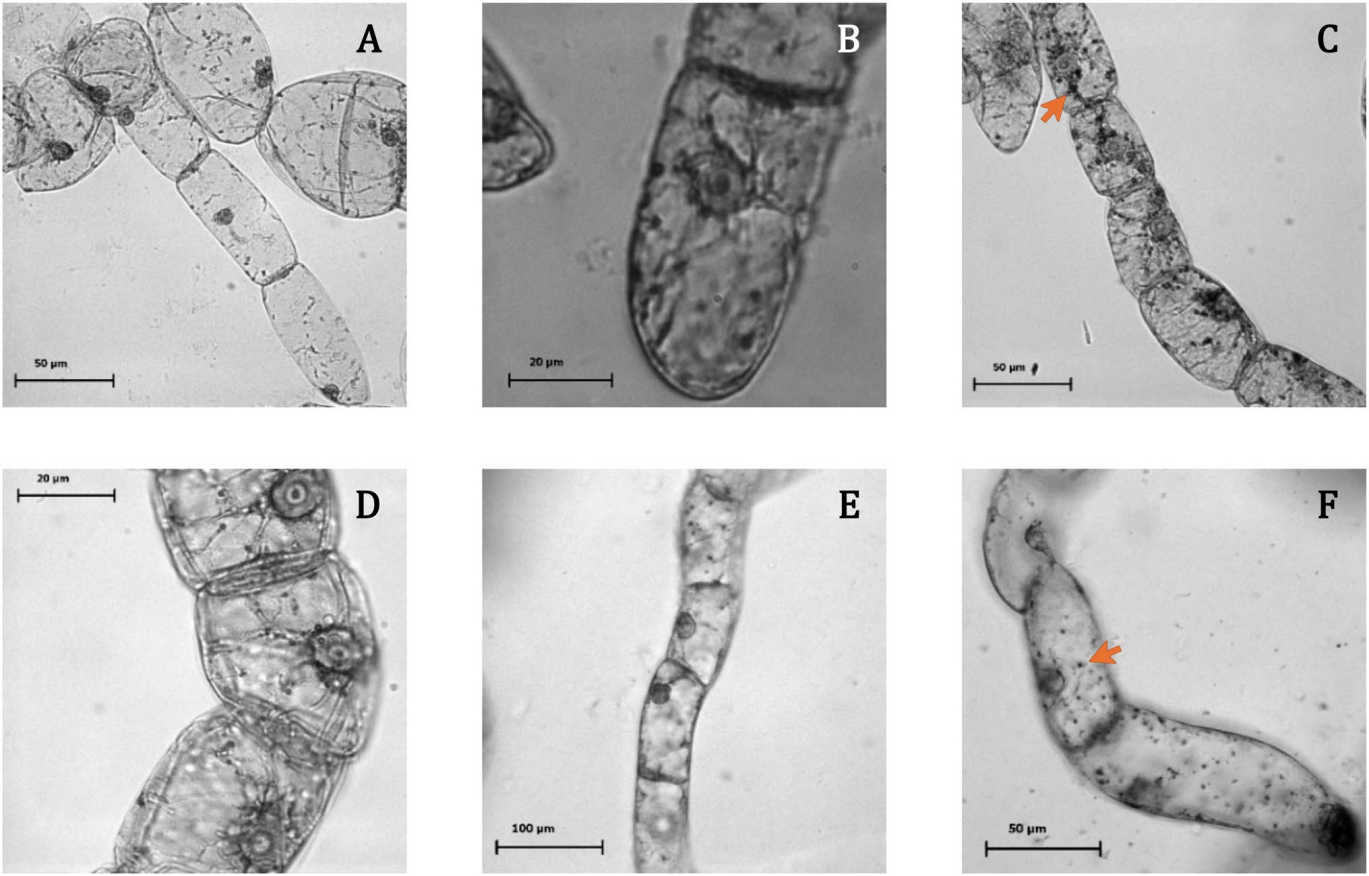
Bright-field images of BY-2 cultured cells. Cells were grown under the following conditions: **A** in standard MS medium, **B** in auxin-deprived MS medium for 24 h, **C, D** in auxin-deprived MS medium supplemented with 0.1 µg.mL^−1^ of Kin for 24 h or **(E, F)** 48 h. **F** Representation of a z-stack of 3 images. Orange arrows indicate the presence of amyloplasts

Lugol’s iodine staining (Sakai et al. 1992; Miyazawa et al. 2002b) confirmed the differentiation of proplastids or etioplasts into amyloplasts, with visible starch granules in cells grown in auxin-deprived medium (Fig. 3). In contrast, WT cells cultured in standard conditions showed no significant staining. Some cells accumulated starch, indicating amyloplast differentiation, while others exhibited weaker staining, suggesting variable levels of starch accumulation.

Carotenoid storage outside of chloroplasts has been observed in algae and non-green tissues of plants, where secondary carotenoids like astaxanthin accumulate in lipid bodies or chromoplasts (Bar et al. 1995; Brackmann et al. 2011; Mateu et al. 2016; Andersen et al. 2021). In our BY-2 cells, iodine staining confirmed that amyloplast differentiation could be induced with the withdrawal of 2,4-D, while kinetin facilitated starch accumulation within these differentiating plastids. These results suggest the presence of plastids in BY-2 cells that may function as compartments for carotenoid accumulation. The very low levels in WT-D cells could reflect a limited development of such storage structures. Theses observation aligns with findings reported in non-photosynthetic tissues, including the orange cauliflower mutant (Li et al. 2001) and carrot root (Kim et al. 2010), where high carotenoid levels appear to trigger chromoplasts differentiation. This can be considered an adaptive response to excess of carotenoids, leading to the formation of sequestration substructures and membrane remodeling (Sun et al. 2018). However, in BY-2 cells, the precise subcellular site of carotenoid accumulation still needs to be clarified.

### Engineered tobacco BY-2 cells display vesicle-type structures

**Fig. 4:**
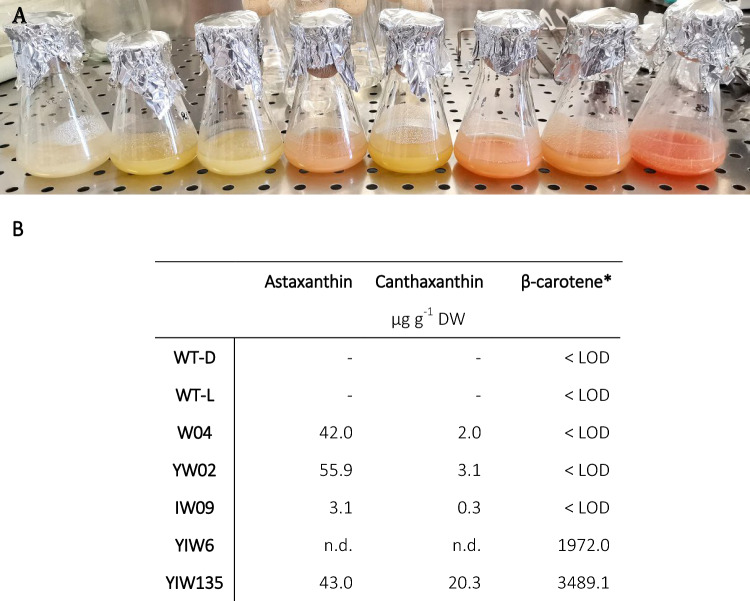
Carotenoid content in tobacco BY-2 cell lines. **A** Representative image of BY-2 cell suspension cultures including WT-L, W04, YW02, IW09 and YIW135. The flasks shown in panel A are intended for illustrative purposes only.** B** Quantification of astaxanthin, canthaxanthin or β-carotene is shown as μg g^−1^ DW (micrograms per gram of dry weight). LOD: limit of detection. *Although β-carotene was not quantified, this pigment is present in all the cell lines since it is the precursor of xanthophylls and ketocarotenoids

To explore the intracellular localization of carotenoids in non-photosynthetic BY-2 cells, five cell lines developed in a previous study (Rebelo et al. 2025; Fig. 4) were selected. Vesicle-like structures were consistently observed in higher amounts in the engineered cells compared to WT-L cells. Given that chloroplasts are a major plastid type involved in carotenoid accumulation, one hypothesis was that these vesicle-like structures could represent developing chloroplasts. However, when examined under fluorescence microscopy, no red autofluorescence characteristic of chlorophyll was detected, suggesting that these structures do not correspond to fully differentiated chloroplasts. This observation is consistent with previous reports indicating that plastids in tobacco BY-2 cells remain undifferentiated and lack the thylakoid membrane organization characteristic of functional chloroplasts (Baginsky et al. 2004). Thus, these vesicle-like structures may correspond to plastid-related organelles or lipidic structures associated with carotenoid storage.

These observations are in line with studies showing the formation of similar structures in response to carotenoid accumulation in *Nicotiana benthamiana* and *Nicotiana tabacum* photosynthetic tissues (Majer et al. 2017; Andersen et al. 2021). In these systems, overaccumulation of phytoene and lycopene in extraplastidial compartments led to the formation of vesicular or crystalline structures. However, when carotenoids accumulated excessively in the cytosol, they were associated with necrosis responses in the leaves (Majer et al. 2017), a phenotype latter mitigated by Andersen and colleagues (2021). Phytoene stored in extraplastidial locations appeared to be less tightly associated with membranes, resulting in improved bioaccessibility. Moreover, lycopene produced in the leaves was shown to form cytosolic crystals, likely after an initial phase of membrane-associated storage, once intracellular concentrations became sufficiently high. Microscopy observations also revealed the formation of a tubular structure that likely corresponds to lycopene crystals (Andersen et al. 2021).

The crystalline structures have been previously reported in other species and are typically found in the chromoplasts of non-green tissues lacking chloroplasts (Maass et al. 2009; Nogueira et al. 2024). In non-green plastids, carotenoid accumulation alone can induce crystal formation, without the developmental programs normally required for chromoplast differentiation. Maass and colleagues showed that in Arabidopsis protoplasts, WT cells contained only unpigmented proplastids, whereas transgenic lines developed orange crystalline particles. Increasing the flux through the carotenoid pathway was sufficient to cause both carotenoid overaccumulation and plastid remodeling, showing that plastids can structurally adapt (Maass et al. 2009). As for chromoplasts, carotenoids are deposited in specialized substructures such as plastoglobules, fibrils, crystals, or proliferated membranes. In tomato fruit, ketocarotenoids accumulate predominantly in crystalline form within plastid membranes and plastoglobules. Using transmission electron microscopy and subchromoplast compartment fractionation, Nogueira and co-workers (Nogueira and co-workers 2024) demonstrated that high carotenoid levels induce remodeling of plastid internal architecture. In the ketocarotenoid-producing lines, plastoglobules were significantly larger and more numerous compared to controls. Whereas lycopene and β-carotene were detected in crystalline form within membranes, only ketocarotenoids were sequestered into plastoglobules, indicating a substrate-specific storage strategy. Plastids can have multiple adaptive mechanisms to cope with the metabolic load imposed by heterologous ketocarotenoid biosynthesis without causing deleterious effects on the host. These results provide evidence that plastid remodeling and specialized sequestration substructures are predictable outcomes of enhanced carotenoid biosynthesis in plant systems.

Based on this principle of plastid adaptations based on metabolic demands, we hypothesized that in BY-2 cells carotenoid accumulation might occur in plastid-related structures, but could also extend into the cytosol, possibly within lipidic structures or membranes. This is supported by the fact that precursors of the carotenoid biosynthetic pathway, such as IPP and DMAPP, are produced both in plastids via the MEP pathway and in the cytosol via the MVA pathway. IPP and DMAPP are then converted into geranyl pyrophosphate, a precursor of phytoene (Rebelo et al. 2020a).

### Intracellular localization of carotenoids confirmed by Raman microscopy

We employed single-cell Raman imaging to determine the intracellular location of carotenoids in the tobacco BY-2 cultured cells. As shown in the previous section, the main carotenoids synthesized by BY-2 cells are β-carotene, astaxanthin, and canthaxanthin. Therefore, the Raman analysis of these three carotenoids (pure standards) was performed to understand the potential spectroscopic differences (e.g., position of the peaks and their relative intensities). Since standard concentrations were below the detection limit of conventional Raman spectroscopy (< 2 mM), SERS was used to overcome this limitation (Stiles et al. 2008; Rodríguez-Lorenzo et al. 2010). GNSs were selected as optical enhancers because they concentrate the local electromagnetic field on the apex of their tips, producing strong Raman signal amplification (Rodríguez-Lorenzo et al. 2009, 2010). TEM and UV–VIS-NIR analysis (Figure S2) revealed a monodisperse distribution in shape and size (79 ± 12 nm) of the GNSs, and the maximum wavelength of the localized surface resonance plasmon in the near-infrared range, which was in resonance with the laser line selected for the analysis (i.e., 785 nm). After functionalization with DDT, the three standards were incubated individually with DDT-GNSs, and subsequently, SERS spectra were acquired using the 785 nm laser excitation line. The presence of DDT improved carotenoid adsorption onto the GNSs surface, forming a more stable self-assembly via hydrophobic interactions, similar to that described for carotenoids incorporated into liposome membranes (Augustynska et al. 2015; Hachlica et al. 2024).

**Fig. 5:**
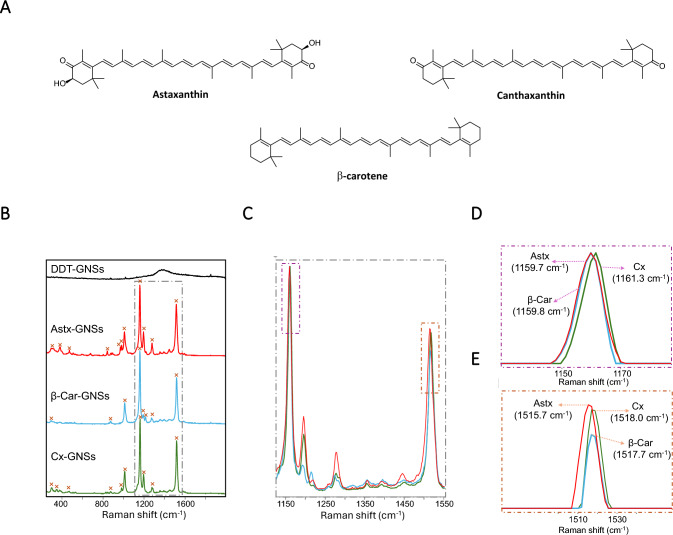
SERS analysis of carotenoid standards. **A** Chemical structure of astaxanthin (Astx), β-carotene (β-Car) and canthaxanthin (Cx). **B** Average SERS spectra of DDT-GNSs alone and in the presence of Carotenoid standards: astaxanthin (red), β-carotene (blue), and canthaxanthin (green). Peaks in each spectrum are marked with “ × ”. **C** Normalized SERS spectra of the carotenoids focused in the spectra windows from 1100 to 1560 cm^−1^, where the two most significant peaks are clearly visualized: at ~ 1160 cm^−1^ (normalized to 1) and ~ 1520 cm^−1^. **D** and **E** show the variation of the position of these two significant peaks as a function of the carotenoid type

It has been reported that polar carotenoids (e.g., xanthophylls ketocarotenoids) typically orient perpendicular to the membrane surface, while non-polar carotenes like β-carotene show a more random orientation, to interact only with the hydrophobic part of the phospholipids (Hachlica et al. 2024). This hypothesis is consistent with the higher variability in the SERS spectra observed in β-carotene, i.e., different orientations with respect to the gold surface, causing a higher difference in the acquired fingerprint due to the surface selection rules in SERS (Le Ru et al. 2011). Figure 5 shows the average SERS spectrum generated from 10 individual measurements per carotenoid, where all spectra display clearly vibrational fingerprint with slight variations in the Raman shift. The SERS spectra had three main peaks: 1009, 1160, and 1516 cm^−1^ for astaxanthin; 1011, 1161, and 1518 cm^−1^ for canthaxanthin; and 1011, 1160, and 1518 cm^−1^ for β-carotene. The peak centered at approximately 1009–1012 cm^−1^ is associated with the stretching vibrations of O–CH₃, CH₃, or C-H groups, corresponding to the υ₃ mode. The peak around 1160–1161 cm^−1^ (υ₂ mode) is attributed to the stretching vibrations of single C–C bonds. The most diagnostic peak for carotenoid identification is the υ₁ mode, which corresponds to the C = C bonds and appears around 1516–1518 cm^−1^ (Llansola-Portoles et al. 2017). These results align with existing literature on Raman vibrational modes for various carotenoids (Withnall et al. 2003; Andreeva and Velitchkova 2005; Barnard and De Waal 2006; Gruszecki et al. 2009; Collins et al. 2011; Jehlička et al. 2014b; Cintə Pinzaru et al. 2015). Importantly, the DDT layer on the gold surface did not interfere with interpreting the vibrational fingerprint of the carotenoids because aliphatic molecules have a much lower cross-section in Raman than conjugated carotenoids (Rodríguez-Lorenzo et al. 2009). As a result, the vibrational features remained clearly discernible.

**Fig. 6:**
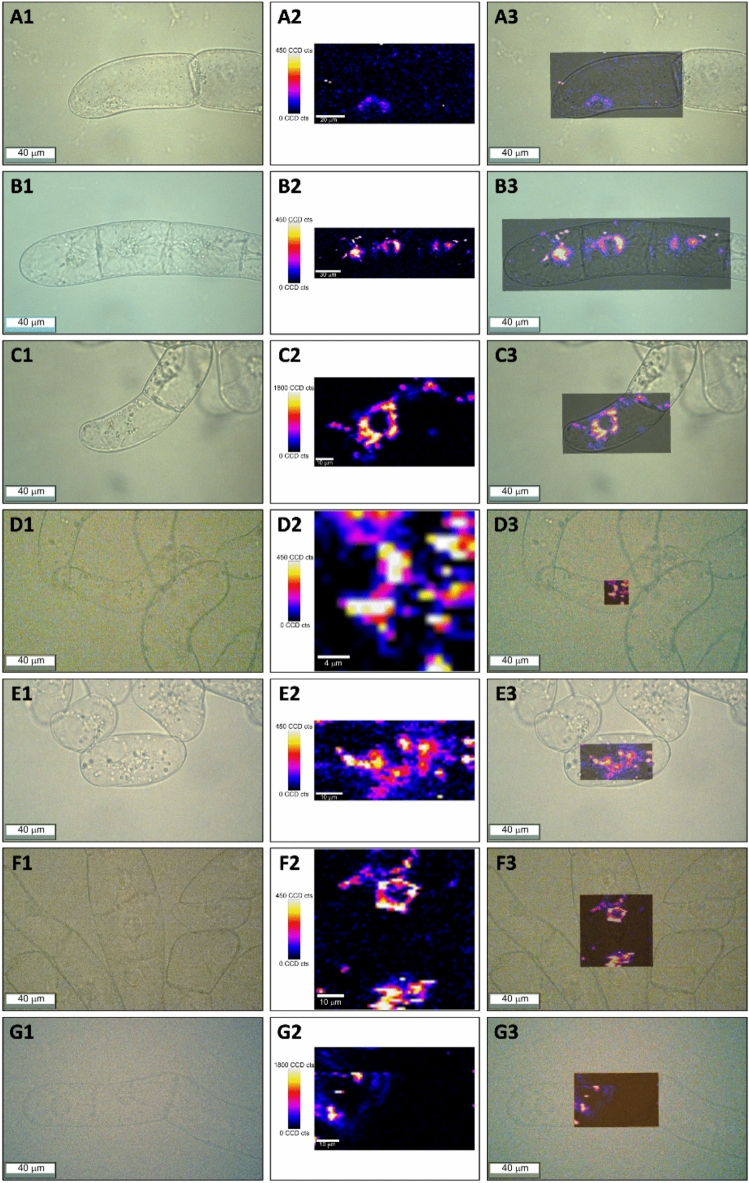
Single-cell Raman imaging of tobacco BY-2 cell lines upon excitation with a 532 nm laser line. Representative cells from WT-D **(A)**, WT-L **(B)**, W04 **(C)**, YW02 **(D)**, IW09 **(E)**, YIW6 **(F)**, and YIW135 **(G).** For each panel, images are presented from left to right as follows: (1) white-light optical images of a selected BY-2 cell, (2) corresponding Raman map (color scale indicates the Raman intensity of the characteristic peak centered at ~ 1520 cm^−1^ for carotenoid standards (Fig. 5) and 1520 – 1530 cm^−1^ for carotenoids into cell lines (Figure S3) within the spectral window 1500–1560 cm^−1^), and (3) overlap image correlating carotenoid distribution with the cellular localization. The Raman map dimension, i.e., number of spectra per map, is different, corresponding to; **A** WT-D: 110 × 55 µm = 4900 spectra, **B** WT-L: 190 × 60 µm = 11,400 spectra, **C** W04: 90 × 50 µm = 4500 spectra, **D** YW02: 20 × 20 µm = 400 spectra, **E** IW09: 60 × 30 µm = 1800 spectra, **F** YIW6: 60 × 60 µm = 3600 spectra, and **G** YIW135: 70 × 45 µm = 3150 spectra. Table S1 shows information on all Raman maps acquired in this work

Taking into account the presence of vesicle-like structures observed in all BY-2 cell lines (see sections “Differentiation of BY-2 amyloplasts led to starch accumulation” and “Engineered tobacco BY-2 cells display vesicle-type structures” for more information) and the SERS dataset of the selected carotenoids, we analyzed the BY-2 cell lines using single-cell Raman imaging. We first identified these structures within the cells and subsequently acquired maps to investigate whether these foci might be associated with carotenoid accumulation. The 532 nm excitation laser line selected offers moderate resonance Raman enhancement and a reduction of the fluorescence background and self-absorption, making it an optimal laser to investigate carotenoids in biological matrices such as cells (Lu et al. 2018). It is worth noting that BY-2 cells are non-photosynthetic and no chlorophyll accumulation was detected in these cells. The absence of fluorescence background was advantageous for Raman measurements, as chlorophyll autofluorescence in photosynthetic cells can overlap with Raman signals and reduce spectral quality. We performed between 4 and 8 maps per cell line, focusing on identifying characteristic peaks, specifically 1160 cm^−1^ and 1520 cm^−1^, corresponding to the stretching mode of C–C and C = C, respectively, as observed in the SERS spectra (Fig. 5). We defined a spectral window between 1500–1560 cm^−1^ to identify the carotenoids in the cell. From the acquired maps, we observed strong carotenoid accumulation in the transgenic lines, while they were barely detectable in WT-D cells (Fig. 6). In the case of WT-D, WT-L and YIW6 cells, the Raman spectra containing characteristic peaks from carotenoids were fitted with the fingerprint of β-carotene, in agreement with the HPLC data (Fig. 4). However, the peak position at 1518 cm^−1^ varied slightly, reflecting the differences in the number of conjugated bonds in the polyene chain and the side groups attached to the carotenoid molecules. Minor shifts, as small as 4 cm^−1^, were also observed in the other two characteristic peaks centered at 1160 cm^−1^ (mode υ₂) and 1012 cm^−1^ (mode υ₃) (Figure S3). These variations likely reflect the presence of structurally similar carotenoids, including isomers and intermediates such as lycopene, echinenone, which may contribute to the small Raman signal shifts observed compared to those of pure standards.

Interestingly, single-cell Raman imaging revealed that carotenoids were primarily localized in subcellular structures resembling vesicles. Raman maps in Fig. 6 show carotenoid accumulation around the nucleus and at various locations of the cytoplasmic membrane. These foci exhibited varying levels of carotenoid accumulation across the metabolically engineered cell lines, with the intensity of Raman signals indicating differences in carotenoid content. The highest intensities were observed in these spherical vesicle-like forms. Given that our BY-2 cells are undifferentiated, non-photosynthetic cells engineered to produce ketocarotenoids, we hypothesize that these cells may have undergone intracellular rearrangements to store these secondary metabolites. The presence of these structures could represent an adaptation mechanism allowing the cells to compartmentalize carotenoids and potentially reduce cytotoxic effects from overaccumulation, though further investigation is required to confirm their nature and role.

### Automated Raman spectra analysis for the identification of carotenoids

The non-destructive Raman microscopy approach enabled us to demonstrate that intracellular vesicle-like structures accumulated carotenoids in BY-2 cells. However, identifying the mixture of carotenoids synthesized for each cell was not possible. On the one hand, SERS analysis was limited to three pure carotenoid standards, while BY-2 cells normally accumulated a variety of carotenoids. This is expected, as the carotenoid biosynthetic pathway is a multi-step route that produces several compounds sequentially (Rebelo et al. 2020a). On the other hand, HPLC analysis demonstrated that among the three standards analyzed by SERS, only β-carotene was detected in YIW6 cells (Fig. 4). In the case of WT-L, the β-carotene levels were low for reliable quantification due to poor signal/noise ratio. Moreover, BY-2 wild-type cells lack the β-carotene ketolase enzyme, which converts β-carotene into canthaxanthin, precursor of astaxanthin. As a result, neither of these ketocarotenoids are present in WT cells. Analysis of Raman maps revealed some variability, making precise identification difficult. To address this, we propose a machine learning method for automatically identifying carotenoids and subsequently discriminating between cell types.

A key step in the algorithm is data preparation for the ML model, as shown in Fig. 1, which includes automatic identification of the k-most important peaks (Iranmehr et al. 2025) in the carotenoid standards dataset (Fig. 5 and Figure S4). It is evident from Fig. 5B and Figure S4 that the Raman spectra of different carotenoids are similar, differing only in the functional groups at the chain ends (Fig. 5A), which result in common characteristic peaks. Despite this overall similarity, individual carotenoids exhibit consistent, quantifiable differences in their spectral features, including the number of peaks, slight shifts in peak positions, differences in relative peak intensities, and variations in peak areas and fine spectral structure. Such differences, while subtle, are statistically distinguishable. To visualize this, we plotted the average spectra for each carotenoid standard and each cell line in Figure S4, which clearly show discernible differences between categories.

In addition, we quantified these differences using intra- and inter-class distances and calculated separation ratios for the carotenoid standards (astaxanthin, β-carotene, and canthaxanthin; Table S4 and details of the method in Supporting Information). The intra-class distance, representing the average variability within each class, ranged from 0.159 to 0.241, while the mean inter-class distance, reflecting separation between class means, ranged from 0.284 to 0.301. The corresponding separation ratios ranged from 1.24 to 1.89, indicating that inter-class variability generally exceeds intra-class variability. Astaxanthin and canthaxanthin showed higher separation ratios (1.56 and 1.89, respectively), suggesting good discriminability, whereas β-carotene exhibited a lower separation ratio (1.24), reflecting slightly closer similarity to the other carotenoids. Overall, these results confirm that despite the structural similarity of the carotenoids, the Raman spectral features contain sufficient subtle differences to enable meaningful class distinction by the neural network (Table S4, Figure S4).

**Fig. 7:**
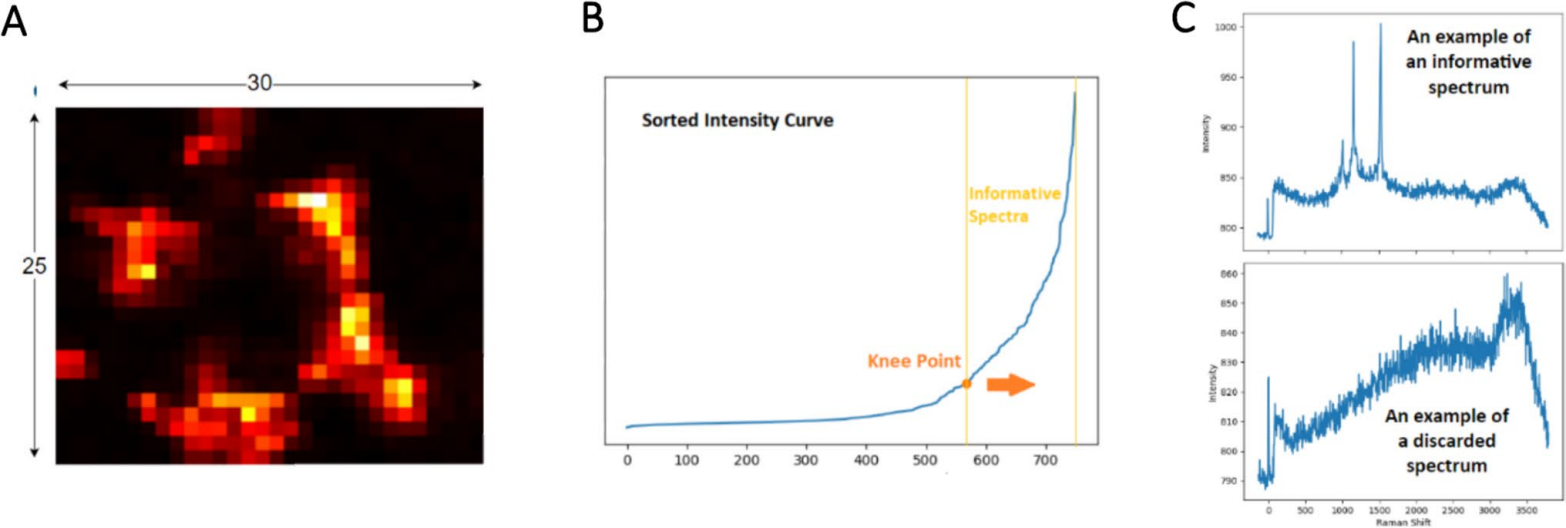
A depiction of clustering informative spectra from non-informative ones in a 30*25 mapping of WT-L cell line. **A** Image of the Raman mapping, where each pixel corresponds to a spectrum. Brighter pixels indicate spectra with more prominent peaks at 1160 cm^−1^ and 1520 cm^−1^. **B** Sorted intensity curve with the identified knee point, used to discard non-informative spectra for subsequent processing. **C** Examples of informative and non-informative spectra, offering a visual reference for typical spectral differences

Another crucial step is clustering plant cell spectra from the Raman mappings into informative and non-informative groups. This step significantly reduces the data volume and accelerates the identification process. This approach was essential because a significant portion of the spectra in the cell lines lacked relevant carotenoid information. Including such data could mislead the machine learning model and unnecessarily prolong the preprocessing time. This process is illustrated for the mapping of BY-2 WT-L cell line (Fig. 7). As detailed in Fig. 1, each spectrum is represented by the sum of intensities at 1160 and 1520 cm^−1^. For each cell line map, these values are placed in a vector, sorted, and the knee point of the sorted intensity curve is identified to cluster the spectra. Spectra located before the knee point (Fig. 7) are deemed non-informative and discarded. Baseline correction (Baek et al. 2015) and spike removal (Whitaker and Hayes 2018) were then applied to the remaining informative spectra before preparing the dataset for training the machine learning model.

To prepare the data for training and evaluating the proposed neural network model all non-noisy spectra from the carotenoid standards dataset were used. For the Raman mapping dataset, we randomly selected half of the informative spectra for each map of each cell line and calculated the average spectrum. This was repeated 10 times, resulting in 10 representative spectra per cell line. Before training the neural network, it was crucial to ensure that all the spectra had the same number of features. When needed, interpolation was applied to resize spectra, followed by feature normalization and data splitting into training and testing sets. For the splitting strategy, Multi-label stratified KFold cross-validation (Sechidis et al. 2011) was employed to evaluate the model in multi-label regression tasks. Multi-label regression tasks involved predicting multiple continuous output variables simultaneously. This technique splits the dataset into multiple folds, ensuring that each fold has a similar distribution of label combinations as the entire dataset. This approach provided a more accurate assessment of the model’s performance by maintaining the balance of labels across training and testing/validation sets. Figure S5 illustrates all the spectra used for training and validating the neural network, including the non-noisy spectra from the carotenoid standards dataset and the representative spectra for each cell line. While multiple strategies exist for generating representative spectra, we employed one of the simplest strategies.

**Fig. 8:**
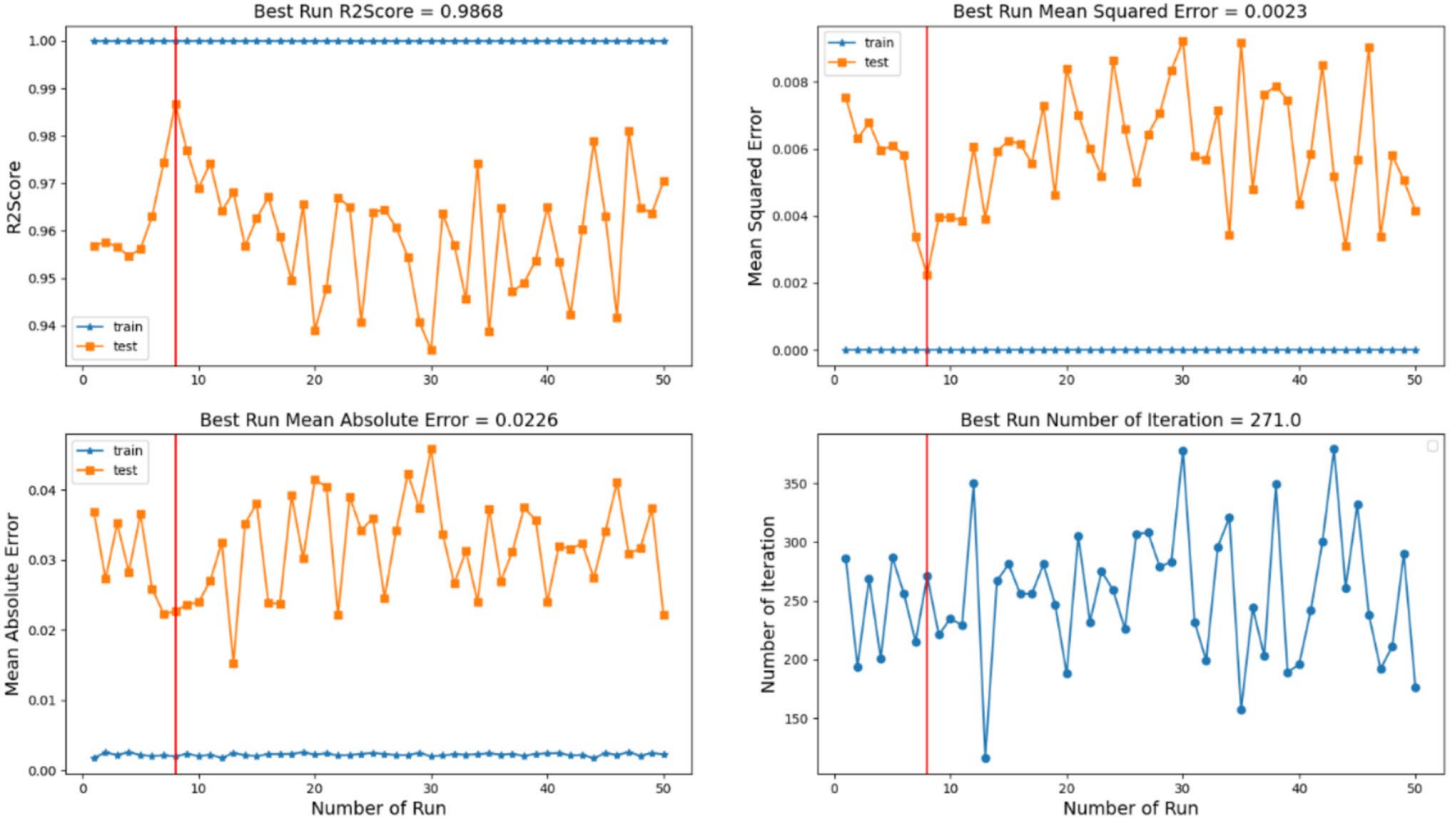
The regression score, MSE, MAE, and number of iteration over 50 runs of the designed neural network (trained with L-BFGS optimizer) for the training and testing data. The best run was shown by a vertical red line

We selected the MLPRegressor neural network model for the prediction of the percentage of carotenoids in cell lines. The closer the true and predicted percentages of carotenoids are to each other, the more accurate the model will be. To train the neural network, we used both the “ADAM” and “L-BFGS” optimizers. L-BFGS is an optimization algorithm that finds the local minimum of an objective function by utilizing both the function values and its gradient. It approximates the Hessian matrix without requiring expensive computations. The model was trained 50 times with each optimizer and performance was assessed using R^2^ Score, MSE, and MAE. As shown in Fig. 8, the best R^2^ Score achieved with the L-BFGS optimizer (Liu and Nocedal 1989) was 0.9868, compared to 0.9216 with the ADAM optimizer (Kingma and Ba, 2014) (see Table 1), indicating superior predictive accuracy and performance.

**Table 1:** Evaluation of the machine learning regressor model on test data using stratified fivefold cross validation. The following tables present the average, standard deviation (SD), and best result of each measure across 50 runs, using both the L-BFGS and ADAM optimizers

Optimizer	L-BFGS			ADAM		
Measure	Mean	SD	Best	Mean	SD	Best
R^2^ Score	0.9566	0.0164	0.9868	0.8823	0.0389	0.9216
MSE	0.0063	0.0023	0.0023	0.0179	0.0058	0.0114
MAE	0.0327	0.0081	0.0226	0.0876	0.0095	0.0771
Number of Iteration	252	41	271	120	25	137

With this work, we developed an automated data processing method to predict the percentages of three carotenoids in BY-2 cell lines using single-cell Raman imaging. The proposed model demonstrated strong performance by accurately quantifying prediction errors using key regression metrics. Employing KFold stratified cross-validation also ensured consistent performance across different data subsets, confirming the model’s stability and generalizability.

## Conclusions

Our study demonstrates that Raman microscopy, coupled with an automated machine learning-based workflow, offers a non-destructive, rapid and label-free approach for identifying carotenoids at the single-cell level in engineered plant cell lines. This approach revealed that vesicle-like intracellular structures were the primary sites of carotenoid accumulation in BY-2 cells. These findings align with previous studies reporting extraplastidial carotenoid storage in *Nicotiana* species, where similar vesicular or crystalline structures were observed in response to phytoene or lycopene overproduction (Majer et al. 2017; Andersen et al. 2021). Moreover, such structures, often cytosolic and lipidic, have also been observed in chromoplasts of non-green tissues and more recently confirmed by TEM in *N. benthamiana* leaves engineered for β-carotene accumulation (Morelli et al. 2024). Our observations reinforce the hypothesis that carotenoid sequestration structures are not only developmentally programmed, but may also be induced by metabolite overaccumulation, even outside plastid compartments. The vesicle-like structures containing carotenoids detected by Raman in BY-2 cells most likely represent plastid-related compartments, such as proplastids, although we cannot exclude the possibility that they correspond to cytosolic lipid droplets or other membrane-bound structures.

Beyond these biological insights, we integrated advanced spectral preprocessing and machine learning to achieve accurate predictions of carotenoid composition without requiring manual intervention. By using peak-driven filtering, our method discards non-informative spectra and retains relevant signals for downstream neural network-based regression. This enables label-free prediction of carotenoid composition directly from single-cell Raman maps, significantly reducing expert workload and minimizing manual bias. The use of machine learning is particularly well suited to analyze the large and complex datasets generated by Raman spectroscopy, enabling the discovery of subtle spectral patterns and improving classification accuracy across biological replicates (Qi et al. 2023). The strong performance of our model demonstrates its potential for high-throughput applications. The advantages of this fully automated and data-aware approach are clear. It significantly reduces human workload, avoids errors associated with manual processing, and enables consistent and scalable carotenoid quantification. Additionally, the pipeline lays a foundation for future research and applications, including real-time analysis, extension to other biomolecules, or integration into high-throughput systems for biomedical and environmental studies. Beyond carotenoid analysis, this workflow can be adapted to other biomolecules or engineered systems, offering a generalizable framework for integrating vibrational spectroscopy with metabolic phenotyping in plant synthetic biology.

## Supplementary Information

Below is the link to the electronic supplementary material.

Supplementary file1 (DOCX 830 KB)

## Data Availability

All data generated or analyzed during this study are included in this published article and its supplementary information.
