# Supplementary material for: Machine learning-assisted single-cell Raman imaging for rapid, sensitive detection and intracellular mapping of carotenoids in plant cell cultures
Source: Plant Cell Rep. 2026 May 23;45(6):173. doi: 10.1007/s00299-026-03858-x (PMC13198516; doi:10.1007/s00299-026-03858-x)
Supplement: Supplementary file 1 — Supplementary file1 (DOCX 830 KB) [file 299_2026_3858_MOESM1_ESM.docx]

## Supplementary information

**Table S1.** Detailed specification of single-cell Raman mapping dataset**.** The table lists the XY dimensions and number of spectra per map. The symbol asterisk (*) in the Map size column indicates the representative map shown in Figure 6, together with the bright-field image of the cell.

| **Cell type** | **No. of maps** | **Map size (x,y) / µm** | **No. spectra** | **No. of informative spectra** |
| --- | --- | --- | --- | --- |
| WT-D | 4 | (70,70) | 4900 | 87 |
|  |  | (110,55)* | 6050 | 154 |
|  |  | (40,60) | 2400 | 327 |
|  |  | (40,70) | 2800 | 343 |
| WT-L | 3 | (190,60)* | 11400 | 728 |
|  |  | (30,25) | 750 | 182 |
|  |  | (40,75) | 3000 | 260 |
| W04 | 4 | (90,50)* | 4500 | 339 |
|  |  | (25,70) | 1750 | 189 |
|  |  | (60,140) | 8400 | 156 |
|  |  | (25,25) | 625 | 79 |
| YW02 | 8 | (80,80) | 6400 | 750 |
|  |  | (40,30) | 1200 | 182 |
|  |  | (100,45) | 4500 | 597 |
|  |  | (110,40) | 4400 | 73 |
|  |  | (20,20)* | 400 | 76 |
|  |  | (20,20) | 400 | 79 |
|  |  | (20,50) | 1000 | 47 |
|  |  | (20,20) | 400 | 0 |
| IW09 | 5 | (45,95) | 4275 | 314 |
|  |  | (85,35) | 2975 | 339 |
|  |  | (60,30)* | 1800 | 86 |
|  |  | (45,45) | 2025 | 80 |
|  |  | (50,75) | 3750 | 446 |
| YIW6 | 7 | (20,20) | 400 | 110 |
|  |  | (60,65) | 3900 | 204 |
|  |  | (30,25) | 750 | 214 |
|  |  | (40,20) | 800 | 58 |
|  |  | (60,60)* | 3600 | 353 |
|  |  | (20,20) | 400 | 42 |
|  |  | (100,100) | 10000 | 226 |
| YIW135 | 5 | (70,45)* | 3150 | 80 |
|  |  | (20,20) | 400 | 86 |
|  |  | (35,35) | 1225 | 40 |
|  |  | (50,50) | 2500 | 330 |
|  |  | (60,10) | 600 | 61 |

**Table S2.** Specification of the carotenoid standards dataset.

| **Carotenoid-standard** | **No. of Raman Spectra** | **No. of informative spectra** |
| --- | --- | --- |
| **Astaxanthin** | 10 | 10 |
| **Canthaxanthin** | 12 | 12 |
| **β-carotene** | 14 | 12 |

**Table S3.** Percentages of existing carotenoids in each cell line.

| **Cell lines** | **Astaxanthin** | **Canthaxanthin** | **β-carotene** |
| --- | --- | --- | --- |
| **WT-D** | 0.0% | 0.0% | 100.0% |
| **WT-L** | 0.0% | 0.0% | 100.0% |
| **YW 02** | 94.8% | 5.2% | 0.0% |
| **IW 09** | 92.5% | 7.5% | 0.0% |
| **W 04** | 95.5% | 4.5% | 0.0% |
| **YIW 135** | 1.2% | 0.6% | 98.2% |
| **YIW 6** | 0.0% | 0.0% | 100.0% |

**Intra-class and inter-class distances and calculated separation ratios for the carotenoid standards**

In addition, we quantified these differences in spectral features using intra-class and inter-class distances and calculated separation ratios for the carotenoid standards (Astaxanthin, β-carotene, and Canthaxanthin, Table S4). The intra-class variability was defined as the average Euclidean distance between individual spectra and their corresponding class mean spectrum, providing a measure of within-class consistency. Specifically, for a given class $k$, the intra-class distance was computed as *Eq* (S1), where $N_{k}$ is the number of spectra in class $k$, $x_{i}^{(k)}$ represents the $i-th$ spectrum in that class, and $\mu^{(k)}$ is the mean spectrum of class $k$.

$d_{intra}^{(k)} = \frac{1}{N_{k}} \sum_{i=1}^{N_{k}} \left\| \left. x_{i}^{(k)}- \mu^{(k)} \right\| \right._{2}$ *Eq* (S1)

Inter-class variability was quantified by computing the Euclidean distances between class mean spectra. The mean inter-class distance for class $k$ was defined using *Eq* (S2), where $K$ is the total number of classes and $\mu^{\left( j \right)}$ denotes the mean spectrum of class $j$.

$d_{inter}^{\left( k \right)} = \frac{1}{K-1} \sum_{\begin{matrix} j=1 \\ j\neq k \end{matrix}}^{K} \left\| \left. \mu^{\left( k \right)}- \mu^{\left( j \right)} \right\| \right._{2}$ *Eq* (S2)

To assess class separability, a separation ratio was calculated as the ratio between inter-class and intra-class distances using *Eq* (S3).

$R^{k}= \frac{d_{inter}^{\left( k \right)}}{d_{intra}^{(k)}}$ *Eq* (S3)

**Table S4.** Intra-class and mean inter-class distances and the separation ratios for astaxanthin, β-carotene, and canthaxanthin. Higher separation ratios indicate stronger spectral discrimination between classes.

| **Class** | **Intra Distance** | **Mean Inter Distance** | **Separation Ratio** |
| --- | --- | --- | --- |
| **Astaxanthin** | 0.182471 | 0.2838 | 1.555 |
| **β-carotene** | 0.240718 | 0.2993 | 1.243 |
| **Canthaxanthin** | 0.159424 | 0.3012 | 1.889 |

| **A** |  | **B** |  | **C** |  |
| --- | --- | --- | --- | --- | --- |
|  | **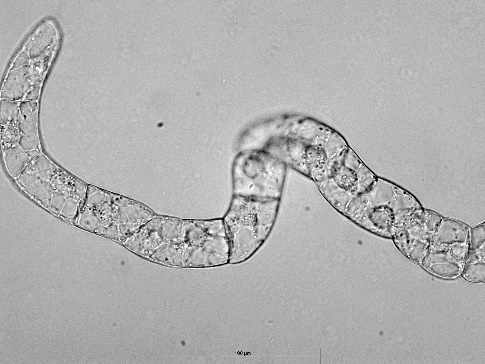** |  | **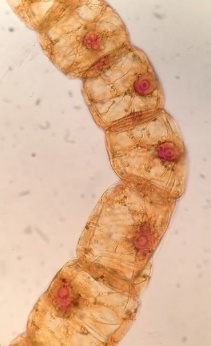** |  | **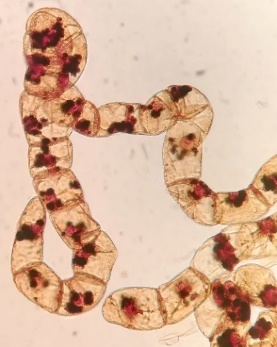** |

**Figure S1.** Bright-field images of wild-type BY-2 cells. **(A)** WT-D cells were grown in standard MS medium. **(B)** WT-L cells were grown in MS medium supplemented with 1 µg mL^-1^ kinetin. **(C)** WT-L cells were transferred to MS medium lacking 2,4-D and supplemented with 1 µg mL^-1^ kinetin. **(B-C)** Cells were stained with Lugol solution to visualize starch accumulation.

| 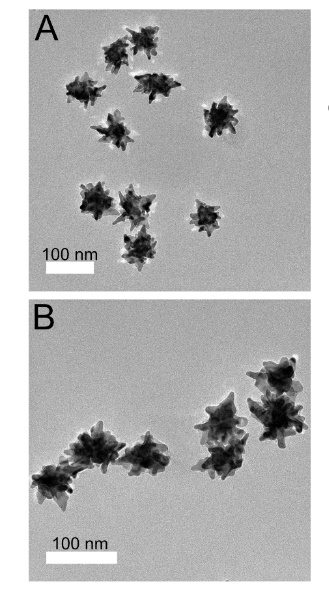 | 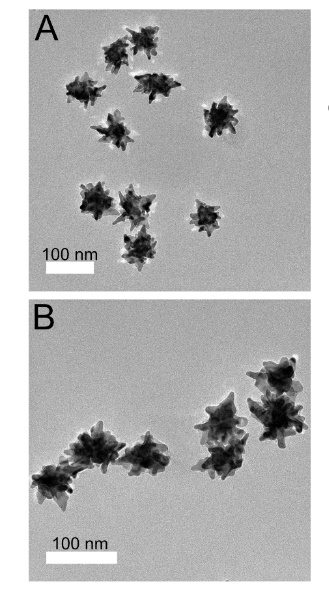 | 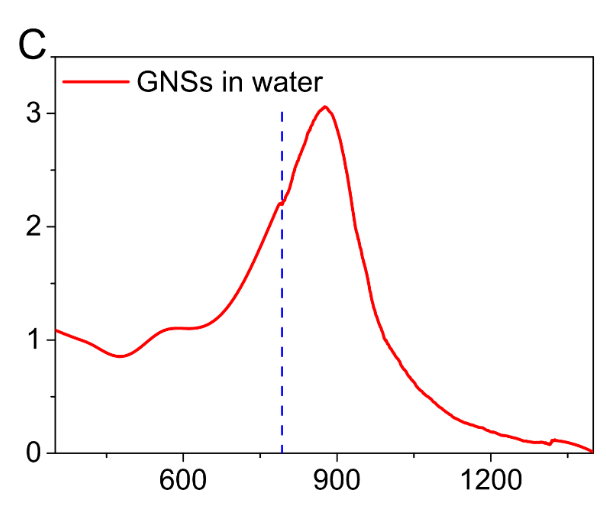 |
| --- | --- | --- |

**Figure S2.** Characterization of gold nanostars (GNSs) by **(A, B)** TEM and **(C)** UV-VIS-NIR. The dashed line in **(C)** indicates the wavelength of the laser line (785 nm) used in the SERS analysis.

**Figure S3.** Average Raman spectra derived from the mapped regions (Figure 6) display the characteristic carotenoid peaks, with the corresponding Raman shifts indicated next to each major band.


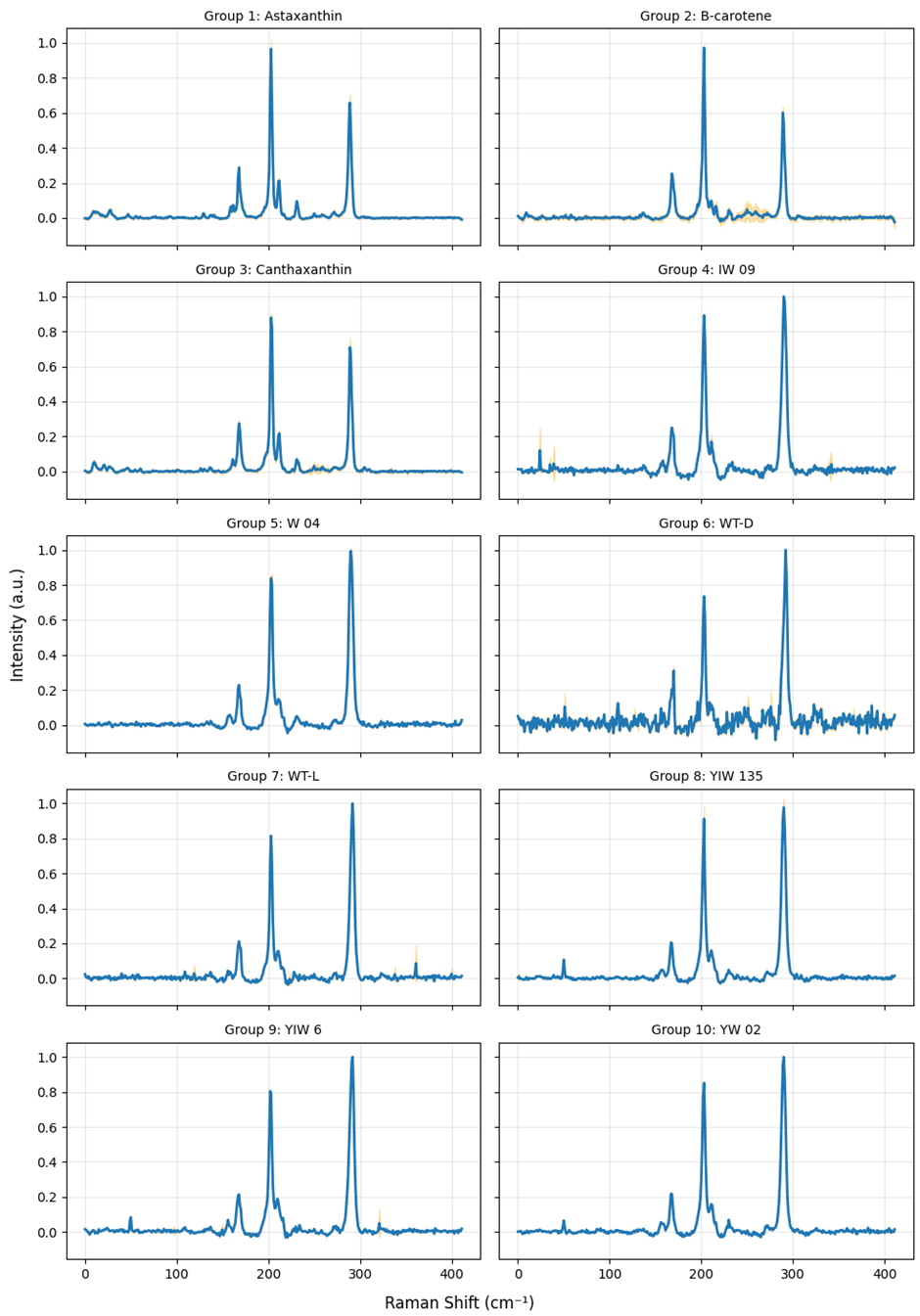


**Figure S4.** Mean Spectra per carotenoid standards and the cell lines with standard deviation.

| **Astaxanthin** | **WT-D** |
| --- | --- |
| ***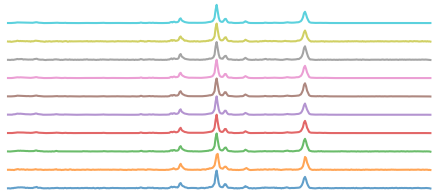*** | ***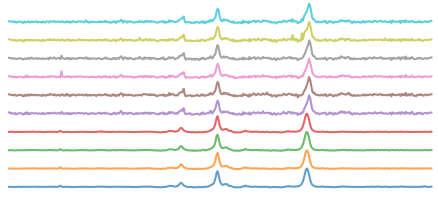*** |
| **Canthaxanthin** | **WT-L** |
| ***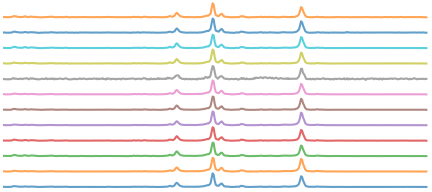*** | ***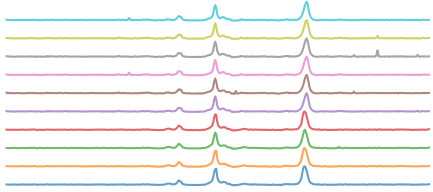*** |
| **Β-carotene** | **W04** |
| ***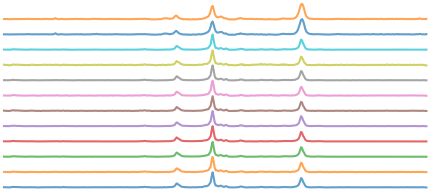*** | ***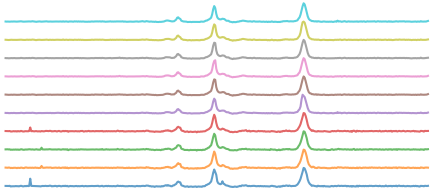*** |
| **YW02** | **IW09** |
| ***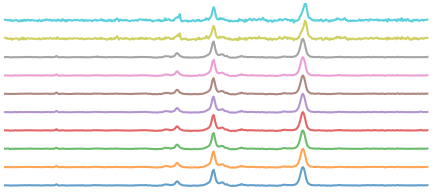*** | ***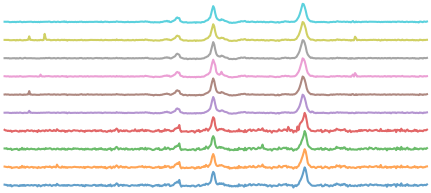*** |
| **YIW6** | **YIW135** |
| ***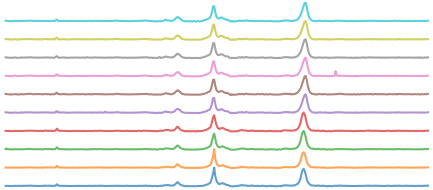*** | ***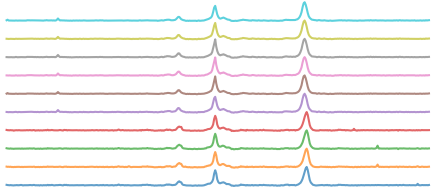*** |

**Figure S5.** Spectra used for training and evaluating the machine learning model. For each cell line, 10 spectra were generated. For each carotenoid standard (astaxanthin, canthaxanthin and β-carotene), only non-noisy spectra were considered.
